# Terahertz time-domain imaging for the examination of gilded wooden artifacts

**DOI:** 10.1038/s41598-024-56913-6

**Published:** 2024-03-15

**Authors:** Edgar Santiago Reyes-Reyes, Ramón Carriles-Jaimes, Emanuele D’Angelo, Saad Nazir, Corinna Ludovica Koch-Dandolo, Falko Kuester, Peter Uhd Jepsen, Enrique Castro-Camus

**Affiliations:** 1 Centro de Investigaciones en Óptica, A.C., Loma del Bosque 115, Lomas del Campestre, 37150 León, GTO Mexico; 2 Department of Electrical and Photonics Engineering, Technical University of Denmark, Kongens Lyngby, 2800 Denmark; 3 Department of Physics and Material Sciences Center, Philipps-Universität Marburg, Renthof 5, 35032 Marburg, Germany; 4 University of Applied Science and Arts of Southern Switzerland, Le Gerre, Via Pobiette 11., 6928 Manno, Switzerland; 5 Department of Structural Engineering, University of California, San Diego 9500, Gilman Dr, La Jolla, CA 92093 USA

**Keywords:** Terahertz optics, Imaging and sensing

## Abstract

Terahertz imaging is unlocking unique capabilities for the analysis of cultural heritage artifacts. This paper uses terahertz time-domain imaging for the study of a gilded wooden artifact, providing a means to perform stratigraphic analysis, yielding information about the composition of the artifact, presence of certain materials identifiable through their THz spectral fingerprint, as well as alterations that have been performed over time. Due to the limited information that is available for many historic artifacts, the data that can be obtained through the presented technique can guide proper stewardship of the artifact, informing its long-term preservation.

## Introduction

Non-destructive and non-invasive imaging techniques are vital for thorough condition assessment and long-term preservation of historic cultural heritage (CH) artifacts. In particular, the ability to evaluate the state of conservation of an artifact, which may include assessment of its structural integrity, presence of deformations, warping, cracking, delamination, as well as identification of original, modified, replaced materials or contaminants that may alter its life cycle or preservation, is critically important. Diagnostic imaging techniques provide important parameters for the development of an artifact - specific documentation, preservation and if required, restoration plan; terahertz (THz) imaging can add unique capabilities for a comprehensive diagnostic imaging methodology.

THz radiation lies in the far infrared part of the electromagnetic spectrum, with a frequency range between 0.3 and 10 THz ($\sim$1–0.03 mm). It can penetrate many optically opaque materials and is non-ionizing; thus, allowing the analysis of the internal structure of various objects without compromising their safety or that of the operator of the instrument. These characteristics make THz techniques attractive for a broad range of applications across a broad range of fields. Over the past two decades, Terahertz time-domain spectroscopy (THz-TDS) has emerged as a promising technique for conservation science, providing a practical method to perform non-invasive and non-destructive analysis of CH artifacts.

In general, THz-TDS systems generate single cycle THz pulses, which, after irradiating the sample, are recorded in the time-domain^1^. The reflected or transmitted waveform allows the optical parameters of the sample to be calculated. For spectroscopic analysis, time-domain signal is Fourier transformed to obtain the spectral distribution, which in THz systems usually has a range of usable frequencies between 0.1 and 3 THz ($\sim$ 3–0.1 mm). Furthermore, several THz systems are equipped with X-Y scanning platforms, allowing the recording of waveforms at specific coordinates. This results in a dataset where a waveform as function of time is recorded for each position across the X–Y plane, forming a spectral image. This technique, known as Terahertz Time-Domain Imaging (THz-TDI), has found wide application in fields such as medicine^2,3^, engineering^4,5^, agronomy^6,7^, architecture^8,9^ among others.

THz-TDS in reflection geometry is ideal for analyzing multi-layered samples; the incident signal is partially reflected at each interface between the layers constituting the sample due to the change in dielectric parameters, this results in a reflected train of pulses that contains the structural information^10–12^. The time between each echo, the *Time-of-Flight* (ToF), is directly related to the spacing between the layers of the sample. This allows THz-TDI to construct cross-sectional images (B-scans). In CH research these images can be used to perform stratigraphic analysis on objects on interest, such as buildings^13^ or wall paintings^14^. Furthermore, since THz radiation is highly reflected by metals, THz-TDI allows the identification of metallic materials, for example gilding in panel painting^15,16^ or painted wood artwork^17^. Moreover, since many molecules manifest strong absorption at THz frequencies, THz-TDS offers an alternative method for identifying certain pigments in artworks^18,19^.

The list of CH objects studied using THz radiation is extensive, including vessels^20^, architectural monuments^21^, wall paintings^22^, mummies^23^, manuscripts^24^, stones^25^, highlighting the utility of THz-TDS as a complementary research technique in conservation science. This paper studies the diagnostics capabilities of THz-TDI in the context of a gilded wooden icon and presents a stratigraphic analysis of areas of interest, in combination with a distribution map of mercury sulfide, a historically significant pigment, widely used in paintings throughout history. It is worth mentioning that the information initially available about the artwork was extremely limited. No historical records provide details about its manufacturing process, materials used, or any interventions it may have undergone in the past. Therefore, the information revealed through this study is relevant and can be used for a proper intervention or restoration in the future.

## Results

An *icon* is a religious artistic piece commonly found in the Orthodox Church which portraits important biblical characters, such as Jesus Christ, Virgin Mary, or saints. Due to the religious significance, it is common to find gold details in specific parts of the work. In terms of construction, icons are typically composed of a wooden support, followed by a preparation layer, gilding, layers of paint and varnish^26^.

**Figure 1:**
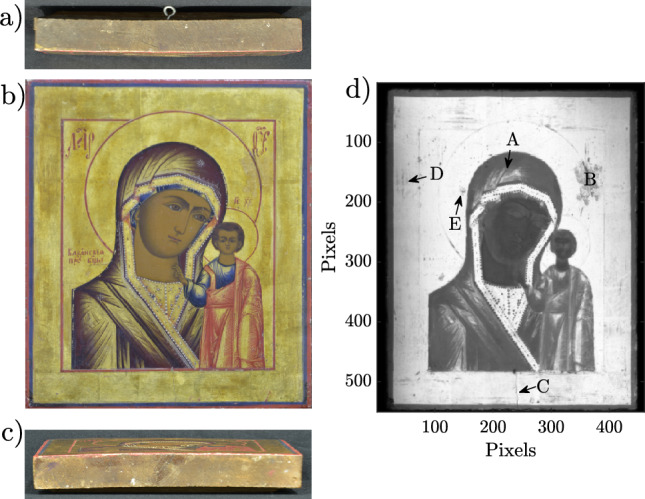
Our Lady of Kazan from unknown artist. (**a**) Upper profile. (**b**) Front view. (**c**) Bottom profile. (**d**) Peak-to-peak image.

In our case, the analyzed painting, shown in Fig. 1b, is a reproduction of the Russian Orthodox icon *Our Lady of Kazan*, by an unknown artist, belonging to a private collection. Painted on a 21.5 $\times$ 27 cm wooden tablet, the icon portrays a bust of the Virgin Mary wearing a golden robe with blue details, with two stars painted on her forehead and the right shoulder. The Virgin Mary is shown carrying infant Jesus, who is wearing blue and red clothing, with only his right arm visible. The icon includes other details, such as the four inscriptions located in the upper corners, to the right of the virgin and above the head of the child, as well as the red halos on the heads of the characters. Traditional gilding was applied, as well as gold details on the clothing. Techniques such as glazes and *impasto* are also found on the work. Unfortunately, information about the period of manufacture or any previous restoration work is not available; however, there are clear signs of, at least, one previous intervention work such as retouching on the left side of the Virgin’s head. In terms of physical deterioration, the icon is clearly bent as can be seen in Fig. 1a and c, which correspond to the top and bottom of the painting respectively. Additionally, there is a crack in the lower part of the painting, as well as small detachments of the different layers that compose the work.

### Peak-to-peak image and time-of-flight

In order to identify the areas where the gold leaf was applied, a peak-to-peak amplitude image was processed and is shown in Fig. 1d). This analysis is done by calculating the difference between the maximum and minimum amplitude in each waveform, which provides information about the reflectance. A strong reflection (white areas on the color scale) is clear evidence of the presence of metallic material, consistent with the use of gold leaf as background in a large area of the icon, as well as in some part of the clothing. Fig. 1d), also reveals the extraordinary artistic skill of the artist, as the characters were painted in areas where the gold leaf had to be removed with extreme precision. The peak-to-peak image also shows the use of a metallic material on clothing, such as the area indicated as (A), which matches all the golden details in the visible image. The difference in intensity of these golden details with respect to the background indicates the use of a metallic paint, typically employed in religious art. Details related with the state of conservation can also be observed, such as the previous restoration work located on the left side of the virgin’s head, indicated by (B), easily distinguishable as a large dark area contrasting with the strong reflection of the background. Furthermore, a large crack in the lower part of the painting, indicated by (C), as well as scratches and small areas where the gold leaf has detached from the surface, for example the areas indicated as (D) and (E) respectively, are visible as small dark spots all over the icon. Moreover, a close visual inspection shows clear signs that suggest that the red frame and halos, along with the inscriptions were painted over the gilding. This fact is supported by the peak-to-peak image, where there is a low contrast in those elements of the icon.

**Figure 2:**
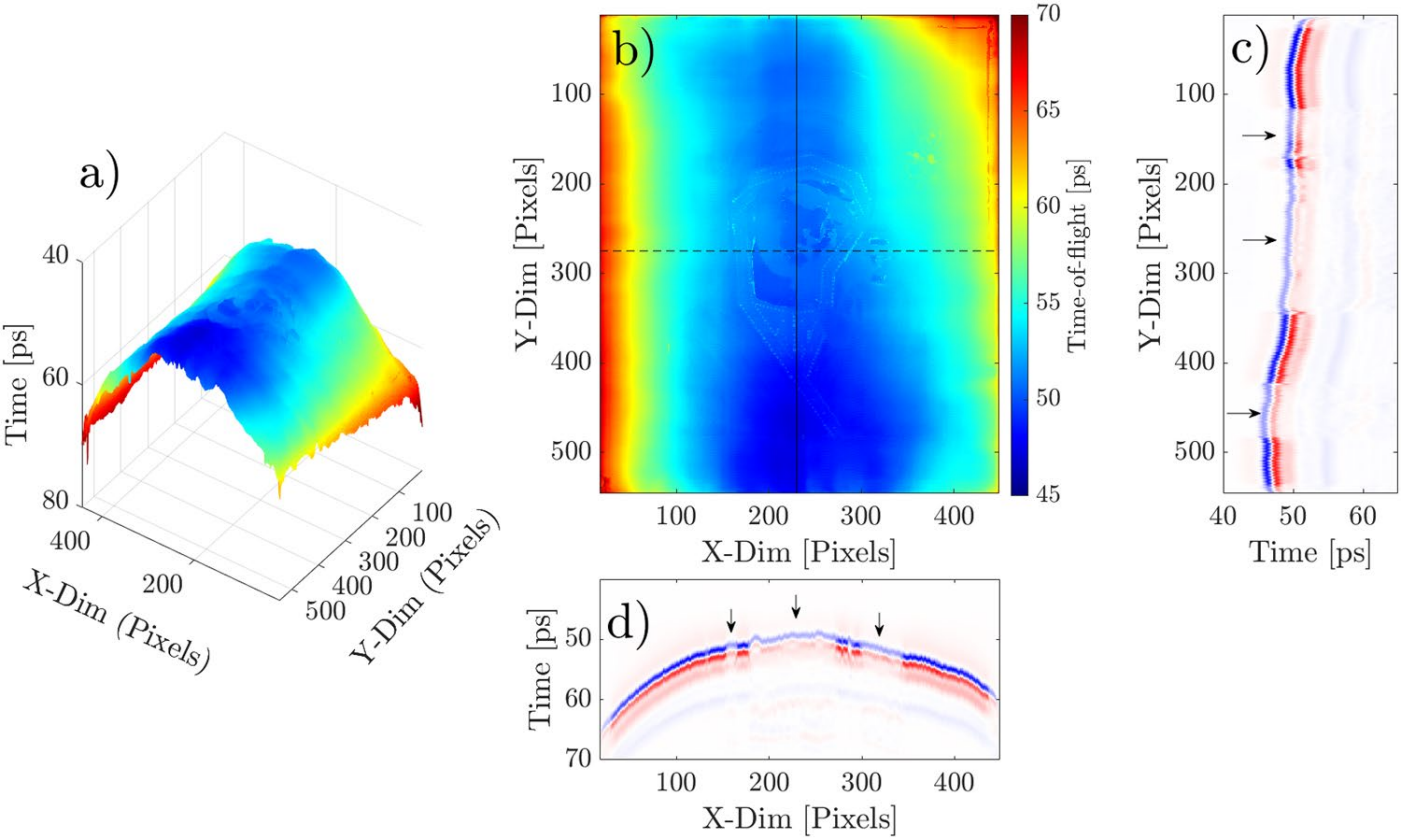
(**a**) 3D Time-of-flight. (**b**) 2D Time-of-Flight. (**c**) B-scan along the y-axis indicated as solid line in the 2D ToF. (**d**) B-scan along the x-axis indicated as dashed line in the 2D ToF. Arrows in B-scans correspond to painted areas where THz radiation penetrates into inner layers on the artwork.

As previously mentioned, a topographic analysis can also be done by THz-TDI examining the ToF, which provides spatial information of the sample, such as surface deformations and reliefs. In order to analyze the state of conservation of the structure, we focused on the time delay of the first echo reflected from the icon, providing topographic information of the surface. Figure 2a), shows the irregular surface of the icon, exhibiting a convex shape along the wooden structure. For a closer analysis, a 2D ToF mapping is shown in Fig. 2b), revealing subtle relief corresponding to the areas of the faces and the impasto applied on the clothing. The significant deformation of the wooden structure is a condition observed in other wooden support artworks and is influenced by different variables, such as the quality and properties of the grain in the wooden panel, the seasoning of the green wood^26^ or the thermo-hygrometric parameters in which the icon is preserved^27,28^.

### Cross-sectional analysis (B-scans)

In order to analyze the internal structure of the icon, B-scans were generated using the raw data of the THz image as a first approach, as shown in Fig. 2c and d, which correspond to the cross sections at the location identified by the solid and dashed line in Fig. 2b, respectively. B-scans confirm the application of gold leaf only in specific areas on the icon, as the THz radiation penetrates the zones indicated by arrows, which correspond to the faces and specific parts of the clothing. This observation provides information about the specific artistic technique used in this work, which can be useful for future conservation efforts. Due to the curvature of the icon, we focused on the analysis of Y-axis B-scans, where the surface remains relatively constant. For a correct multi-layer analysis, a sparse deconvolution algorithm was applied to the raw data to correctly identify the echoes reflected at each interface^29^. Waveforms were normalized to obtain a better contrast on B-scans images.

**Figure 3:**
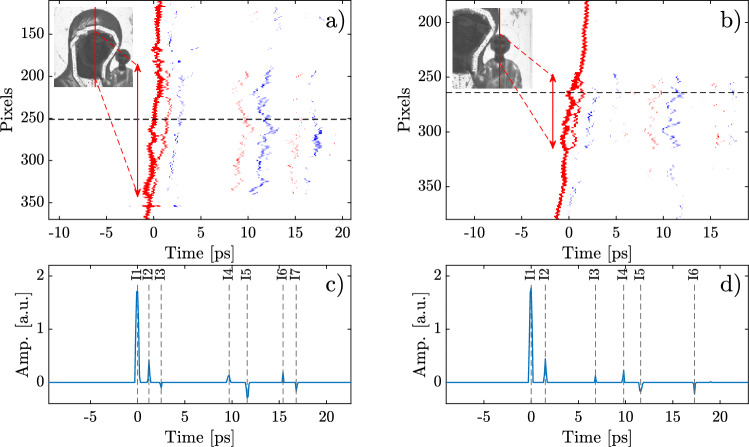
(**a**, **b**) Deconvolved and normalized B-scans. The red line in the insets indicate where the B-scans were measured. (**c**, **d)** Processed waveforms corresponding to pixels 251 and 264, from (**a**) and (**b**) B-scans, respectively, indicated as dashed lines. Interface labeled as I2 is only present in areas corresponding to faces and necks, as shown by the B-scans in the zones indicated by the red arrows. These additional interface can clearly be seen next to the first echo, corresponding to the surface of the icon.

Figure 3a and b show two deconvolved and normalized B-scans, corresponding to the solid red lines in the inset images (pixels 236 and 320 on the X-axis), which include the faces and clothing of the Virgin and the child, respectively. In the color scale, positive peaks are indicated by red pixels, while negative peaks are indicated by blue pixels. These zones exhibit a complex multilayer structure, as shown by the waveforms in Fig. 3c and d, corresponding to pixels Y = 251 and 264, respectively. The change in polarity of the reflected echoes is caused by the difference in refractive indices of the materials at the corresponding interface. We identified up to seven interfaces, or six layers, composing the analyzed B-scans. Furthermore, we found that interface identified as “I2” is only present in specific areas of the faces and necks, as shown in the B-scans. This finding is consistent with what is observed in Fig. 2b, where it is possible to appreciate the relief on the areas of the face and neck of the characters, same areas where interface I2 was found. Due to the presence of an additional interface, the thickness in these areas is higher than in the rest of the artwork.

Considering that the depth penetration in reflection geometry can be calculated as $\Delta x = c \Delta t/2 n$, where $\Delta t$ is the time between the first and the last recorded echo, *c* is the speed of light, and *n* is the refractive index in the THz regime, taking $\Delta t$ from the waveform in Fig. 3c, and assuming an average refractive index of 1.5 for the materials used in the manufacture of the icon, the distance between the surface and the last interface is $\Delta x \approx 1.7$ mm,

### Mercury sulfide identification

Another important aspect that was previously mentioned is the ability to identify certain compounds by THz spectroscopy, since some minerals exhibit absorption bands in the THz range. One such pigment is mercury sulfide (HgS), also know as *vermilion* or *cinnabar*, which exhibits an absorption peak centered 1.14 THz^30^. This red pigment has been widely used in various cultures over time due to its appropriate characteristics for painting, such as adhesion, bright color and low hardness, making it easy to grind^31^. This pigment was found to be part of the pigments used in the icon, with no evidence of the use of any other pigment recognizable by THz-TDS.

**Figure 4:**
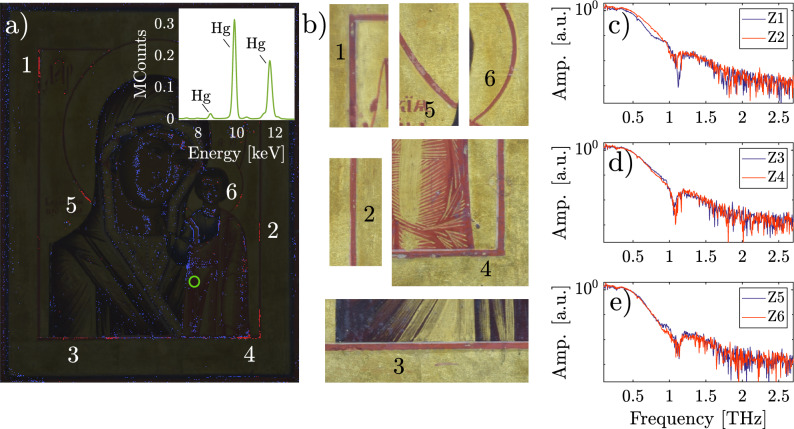
(**a**) Mercury sulfide detected in the painting, indicated by red pixels; six zones were clearly identified. Blue pixels correspond to spectra were vermilion possibly be present. Inset shows the XRF emission spectrum taken from the green circle. (**b**) Photographs corresponding to zones 1–6, where mercury sulfide was detected. (**c**–**e**) Spectra of waveforms from the six zones. The characteristic absorption peak of mercury sulfide can be clearly seen at 1.14 THz.

With the aim of localizing the areas where vermilion was applied on the icon, we used an algorithm based on the identification of its absorption peak. However, in some spectra, the noise in the frequency domain extends to the vermilion absorption peak, leading to an non-reliable detection of the pigment. For a proper detection of vermilion, we first apply an algorithm for estimating the pixel-by-pixel usable bandwidth over the entire THz image^32^; then we performed a visual inspection of each spectrum to validate the presence of absorption peak of vermilion within the noise. This methodology not only allowed us to correctly identify pixels where the absorption peak is present, but also pixels where vermilion could possibly be present (an absorption peak can be seen but not clearly above the noise level), resulting in Fig. 4a.

Blue pixels on Fig. 4a, denote positions where the algorithm detected the presence of vermilion, however, the poor signal-to-noise ratio extending below 1.45 THz make the measurement inconclusive for these pixels. Yet, X-ray fluorescence (XRF) measurements were performed on a reddish area of the children’s robes, indicated by the green circle in Fig. 4a, near to clustering of blue pixels. The XRF emission spectrum revealed the presence of mercury in this specific area, as shown in the inset. This finding would be related with the application of mercury sulfide to the icon. In addition, the algorithm detected the absorption peak of vermilion in other parts of the icon, including the face, clothing and some red details, which are indicated by red pixels in Fig. 4a. And it was, therefore, possible to confirm the presence of vermilion in six zones corresponding to specific parts of the frame and halos which are visibly reddish. The spectra corresponding to these six zones are shown in Fig. 4c–e, where it is easy to distinguish the absorption peak of vermilion. The algorithm also inconclusively suggested the presence of vermilion on the faces but this is inconsistent with the XRF measurements.

Given that there are no visible differences between the mercury sulfide-based pigment and the reddish pigment used in the rest of the icon, this analysis is of special interest since THz-TDI allowed us to differentiate between the two red pigments used in the work. Furthermore, another interesting result is the detection of vermilion only in small areas within the halos and frame, as illustrated in Fig. 4a. The regions where the HgS spectrum was identified resemble retouches applied with this pigment. Possibly, in addition to the gilding leaf intervention, certain areas were selectively treated with vermilion to recreate specific elements of the artwork.

## Discussion and conclusions

In this study, we have successfully used THz-TDS in order to obtain relevant information about the icon ’Our Lady of Kazan’, whose manufacture and restoration history remain unknown. By THz-TDI, we conducted a morphological examination that revealed a physical deformation in the wooden panel, a common condition in wooden artworks. Contrary to common practice, our measurements show that not all the support was covered with gold leaf, only specific parts. We also identified the presence of a metallic paint, presumably gold, applied as ornaments on the clothing. Moreover, we observed areas with detached gilding, cracks and evidence of previous restoration work. Additionally, in areas with non-metallic surface, we were able to perform a stratigraphic study, identifying up to six layers comprising the artwork and detecting an additional interface in specific facial zones. Furthermore, through the identification of the absorption peak of vermilion, we constructed a pigment map, allowing us to map-out areas of restoration work where this pigment had been applied, despite not being originally part of the artwork. This investigation also shows the applicability of generating maps of pigments using safe sources of radiation, such as THz waves. These sources have the same potential to identify certain pigments as XRF measurements confirm, but without the risk of inducing defects in pigments generated by exposure to ionizing radiation.

This finding contributes pertinent details regarding the historical modifications made to the piece. It should be noted that the information obtained in this research, including details such as the structural deformation of the wooden support, the use of HgS and metallic materials, along with the technique applied in the gilding process, can prove valuable for future interventions on the icon, as it provides conservators and restorers a more comprehensive understanding of the artwork. This study exemplifies the contributions of THz spectroscopy on the field of art conservation by revealing hidden details and detecting restoration works that no other non-destructive techniques can. The integration of THz technology alongside other non-invasive methods such as Raman spectroscopy, Fourier Transform Infrared spectroscopy or UV-Vis spectroscopy for identification of chemical compounds, or even techniques using ionizing radiation such as macro XRF for chemical mapping, marks a crucial advancement in art conservation and heritage research.

## Methods

### Terahertz imaging

**Figure 5:**
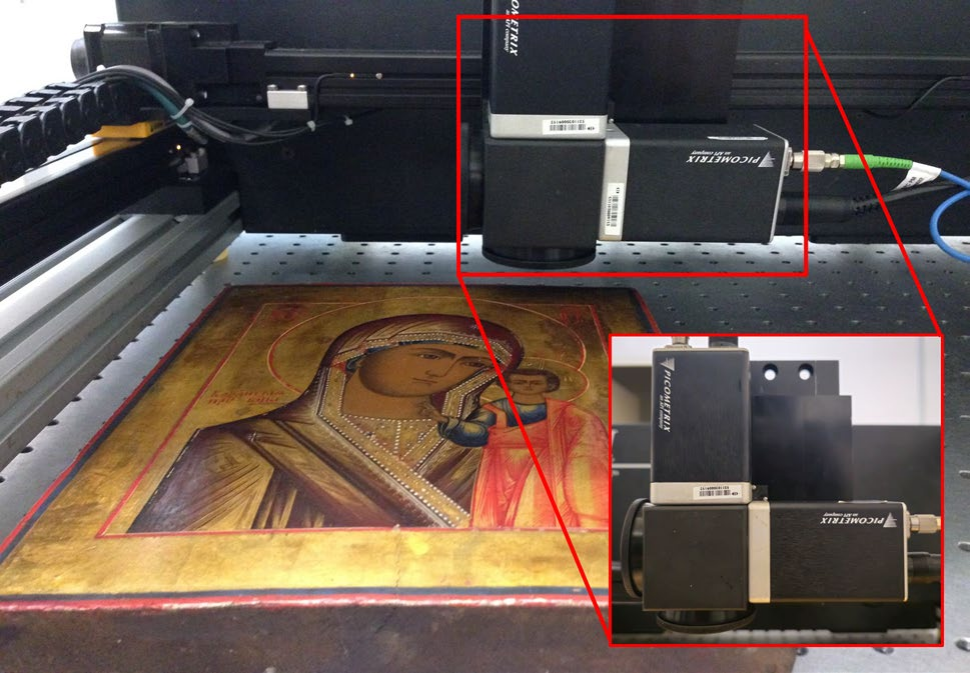
API-Teragauge system with photoconductive transceiver head for measurements in reflection geometry.

The entire icon was scanned using a API-Teragauge TDS system at LANCYTT, Leon, Mexico, which consist of a femtosecond fiber laser coupled with a photoconductive transceiver head, generating THz pulses measured over a temporal window of 160 ps with a resolution of 0.1 ps. The terahertz system is shown in Fig. 5 In the frequency domain, the usable THz bandwidth was between aproximately 0.1–1.8 THz with some fluctuations depending on each measurement. The transceiver system was mounted on a X–Y platform stage to perform the image recording in reflection geometry at normal incidence. The icon was scanned generating a 550 $\times$ 460 pixel spectral image, with a pixel spacing of 0.5 mm.

### Sparse deconvolution

Since the echoes reflected from interfaces can be fully or partially overlapping due to the thickness of the sample layers, a deconvolution algorithm was used. In the time-domain, the reflected THz signal *y*(*t*) can be described as the convolution in the Fourier sense of the incident THz pulse *h*(*t*) with the impulse response function *f*(*t*), which contains information of the sample, following $y(t)=f(t) \circledast h(t)$.

The aim of a deconvolution process is to retrieve *f*(*t*). Since *h*(*t*) is the reference signal in our analysis and *y*(*t*) is the THz signal recorded from a sample, sparse deconvolution calculate *f*(*t*) by assuming that *f*(*t*) is non-zero only at specific locations, approximating *y*(*t*) to $f(t) \circledast h(t)$. The algorithm for sparse deconvolution applied in this work is based on the method introduced by Dong et al.^29^. In our processing, an offset correction on the time-domain data was applied. Additionally, in order to remove noise at low and high frequency, spectra were filtered using a butterworth filter. As a result, we obtained an approximation of Dirac-$\delta$ train, as shown in Fig. 3c and d.

### Pigment mapping

The algorithm for pigment mapping is based on identifying the absorption peak of vermilion by calculating the ratio between the area under the spectrum $\tilde{E}_{sam} (f)$ and *A*(*f*) which is an exponential interpolation of the spectrum. The integral is evaluated over a frequency range between $f_a=1.01$ THz and $f_b=1.27$ THz, Therefore,

$$\begin{aligned} Q=\frac{\int ^{{f}_b}_{{f}_a} A (f)\, \textrm{d}f}{\int ^{{f}_b}_{{f}_a} \tilde{E}_{sam} (f)\, \textrm{d}f}, \end{aligned}$$

gives us a measure of the presence of vermilion.

## Data Availability

The datasets and scripts used and/or analysed during the current study are available from the corresponding author on reasonable request.
